# *Brachypodium distachyon* as a model for defining the allergen potential of non-prolamin proteins

**DOI:** 10.1007/s10142-012-0294-z

**Published:** 2012-08-30

**Authors:** A. Juhász, Gy Gell, E. Sebestyén, R. Haraszi, L. Tamás, E. Balázs

**Affiliations:** 1 Applied Genomics Department, Agricultural Institute, Centre for Agricultural Research, Hungarian Academy of Sciences, Brunszvik 2, Martonvásár, 2462 Hungary; 2 Universitat Pompeu Fabra, Dr. Aiguader 88, 08003 Barcelona, Spain; 3 Institute for Reference Materials and Measurements, European Commission Directorate General Joint Research Centre, Retieseweg 111, Geel, 2440 Belgium; 4 Department of Plant Physiology and Molecular Plant Biology, Eötvös Loránd University, Pázmány P. stny. 1/A, Budapest, 1117 Hungary

**Keywords:** *Brachypodium*, Wheat allergy, Immunoinformatics, Non-prolamins

## Abstract

Epitope databases and the protein sequences of published plant genomes are suitable to identify some of the proteins causing food allergies and sensitivities. *Brachypodium distachyon*, a diploid wild grass with a sequenced genome and low prolamin content, is the closest relative of the allergen cereals, such as wheat or barley. Using the *Brachypodium* genome sequence, a workflow has been developed to identify potentially harmful proteins which may cause either celiac disease or wheat allergy-related symptoms. Seed tissue-specific expression of the potential allergens has been determined, and intact epitopes following an in silico digestion with several endopeptidases have been identified. Molecular function of allergen proteins has been evaluated using Gene Ontology terms. Biologically overrepresented proteins and potentially allergen protein families have been identified.

**Electronic supplementary material:**

The online version of this article (doi:10.1007/s10142-012-0294-z) contains supplementary material, which is available to authorized users.

## Introduction

Cereal sensitivity is a complex phenotype, and its proper analysis is far from complete (Weichel et al. 2006). Wheat prolamins are considered as key players in the formulation of different wheat-related health problems, such as the autoimmune response in patients suffering from celiac disease (CD) or different categories of wheat allergies (WA; e.g. Mittag et al. 2004). Alpha and gamma gliadins are the main protein families responsible for celiac disease (e.g. Chand and Mihas 2006; Catassi and Fasano 2008). These proteins contain a number of T cell stimulatory epitopes either in their repetitive regions (Arentz-Hansen et al. 2000, 2002; Shan et al. 2002) or elsewhere (Van de Wal et al. 1998; Mantzaris and Jewell 1991).

Wheat allergy is an extremely diverse group of wheat sensitivities with various symptoms caused by a number of possible allergens. Omega gliadins are the main contributors of symptoms in wheat-dependent exercise-induced anaphylaxis (Laurière et al. 2007). Sulphur-rich prolamins, such as alpha gliadins and low molecular weight glutenins also contribute to wheat allergies. Other members of wheat prolamin superfamily, such as alpha-amylase inhibitors (Deponte et al. 1976; Gomez et al. 1990; Weiss et al. 1993; Amano et al. 1998; Kusaba-Nakayama et al. 2000) or non-specific lipid transfer proteins may contain epitopes that are related to different allergic symptoms, including baker’s asthma. The effect of allergens is not exclusively related to the prolamin superfamily, although most of the methods and clinical tests are focusing on the identification of proteins of the prolamin superfamily. There are some additional proteins with potentially detrimental effects for patients suffering mainly from wheat allergies. Wheat non-prolamins that have been proven to act as allergens include wheat germ agglutinin, wheatwins (pathogenesis-related proteins of the PR-4 family, Muthukrishnan et al. 2001; Altenbach et al. 2007), triosephosphate isomerase, glycerinaldehyde-3-phosphate dehydrogenases, acyl-CoA oxidase, fructose bisphosphate aldolase and triosephosphate isomerase (Tatham and Shewry 2008; Salcedo et al. 2011). Twelve different IgE-binding protein sequences, such as thioredoxin (cross-reactive allergens), a predicted high mobility protein, a predicted leucine-rich repeat protein, a beta-purothionin precursor and the serine carboxypeptidase II were also identified using Western blotting against patients’ sera (Sampedro and Cosgrove 2005; Weichel et al. 2006).

A more diverse range of food proteins may be allergenic due to sensitization by inhalation (Mills et al. 2003). Cross reactivity caused by epitopes primarily found in pollens results in respiratory allergies, such as baker’s asthma or hay fewer (Donovan and Baldo 1990). The main allergens that have been linked with baker’s asthma include members of the alpha-amylase and trypsin inhibitor family, peroxidase, thaumatin-like proteins, serine protease inhibitors (serpins) and lipid transfer proteins (Salcedo et al. 2011). Thioredoxin is in this category but can also decrease allergenicity at the same time (Buchanan et al. 1997). Further IgE-binding proteins, such as a zinc-ion-binding protein with transcription factor activity, have been identified by De Angelis et al. (2007).

The diversity and number of these proteins as well as their effects compared to prolamin proteins are not clarified so far. The relatively small number of expressed wheat seed proteins found in the protein databases makes it difficult to get a complete overview of the allergen characteristics of the wheat seed. Whole genome sequencing projects carried out on cereals, such as wheat or barley, will facilitate the efforts to reveal the complexity of genetic effects of cereal allergies. So far, *Brachypodium distachyon* serves as the evolutionary closest sequenced genome to *Triticeae* (The International Brachypodium Initiative 2010).

There are three subfamilies of grasses, the *Ehrhartoideae* (rice), the *Panicoideae* (maize, sorghum, sugar cane and millet) and the *Pooideae* (wheat, barley and cool season forage grasses) that diverged from a common ancestor 50–70 million years ago (Bolot et al. 2009). *B. distachyon* is a small temperate grass endemic to the Mediterranean and Middle East, with all the attributes needed to be a model organism including simple growth requirements, short lifecycle, small genome (~300 Mbp) and self-fertility (Draper et al. 2001). The haploid genome size of diploid *Brachypodium* is approximately twice the size of *Arabidopsis* (Bennett and Leitch 2005; Vogel et al. 2006). Thus, *Brachypodium* possesses one of the smallest genomes of any grass and is suitable for both functional and structural genomic research. *Brachypodium*, as the closest relative to wheat or barley due to its genome, is being sequenced and is a suitable base to develop a workflow to identify and classify proteins that are responsible for wheat-related disorders such as celiac disease or wheat allergy.

The slightly longer and slender seeds of the *Brachypodium* differ significantly from those of members of the *Triticeae* both in grain development and composition, and due to a number of other differences, *B. distachyon* is considered an evolutionary intermediate between rice, oat and the *Triticeae* genus. The endosperm is about 75 % of the dehulled seed weight, with lower starch content and thicker cell walls than wheat seeds (Opanowicz et al. 2011). Protein content of the *B. distachyon* seed is about 17 % of the total dry weight, and as such, *Brachypodium* belongs to cereals with high protein content (Guillon et al. 2011). The seed protein composition observed in *B. distachyon* shows some differences compared to the wheat grain proteome. The main storage proteins are globulin type similar to oat and rice (Larré et al. 2010). The number of expressed prolamins—including the avenin-like and gliadin-like prolamins—is much lower compared to wheat and barley, where prolamins serve as the main nutrient reservoirs in the seed (Hands and Drea 2012).

Immunoinformatics or computational immunology provides a tool to predict the immunogenicity of proteins and to determine potential epitopes or design vaccines. With the ongoing sequencing projects in wheat and barley (Feuillet et al. 2011), the potential allergen behaviour of seed proteins will be feasible to determine. Due to its low prolamin content, *B. distachyon* serves as a platform to study allergen potential of non-prolamin proteins. In this manuscript, the *B.distachyon* genome sequence is utilized to map the allergen potential of this model material and evaluate its possible application in human diet as supplementary food source.

## Materials and methods

Immune Epitope Database (IEDB; www.immunoepitope.org) was used to collect and analyse epitope entries of *Pooideae*, the subfamily of *Poaceae.* This subfamily includes all species relevant in terms of cereal-related food allergies and also the genus *Brachypodium*. The epitope hits were filtered against *Homo sapiens* as host organism, and epitopes reported to be pollen allergens were excluded from the analyses. Only linear epitopes causing any kind of wheat allergies or celiac disease have been involved in the study. Epitope peptide sequences have been mapped onto *B. distachyon* predicted protein sequence database (version 1.2) using Epitope Conservancy Analysis tool at IEDB (Bui et al. 2007). Epitope conservancy was calculated at 100 % sequence identity threshold, based on proteins containing peptide sequences identical to identified allergic epitope peptides.

The tBlastN algorithm (Altschul et al. 1990) was used to identify potential allergen proteins expressed in the seed. Translated developing *Brachypodiun* EST database was searched against the potential allergen proteins, and hits with over 98 % sequence homology have been accepted. The single cDNA reads originated from *Brachypodium* seeds that ranged in maturity from anthesis to ripened stage. Rice Genome Browser (MSU Rice Genome Annotation Release 7, Ouyang et al. 2007) and 25 DPA rice endosperm and 25 DPA rice embryo libraries (http://rice.plantbiology.msu.edu/cgi-bin/gbrowse/rice/) were used to check whether rice proteins that were found to be homologous to the annotated *Brachypodium* proteins are expressed in mature rice seed.

To simulate gastrointestinal mechanisms, proteins have been analysed to find potential cleavage sites using the Expasy PeptideCutter tool (Gasteiger et al. 2005). Endopeptidases such as trypsin, pepsin (pH 1.3) and chymotrypsin were involved simultaneously in the in silico digestion analysis, and epitopes that were resistant to the enzymatic cleavage have been identified.

Gene Ontology (GO) terms were assigned to individual proteins using the v1.0 Jan 2009 annotations of the *Brachypodium* genome from the Gramene database (http://www.brachypodium.org/). The Biological Networks Gene Ontology tool (BiNGO) (Maere et al. 2005) was used to find protein families with significantly overrepresented Gene Ontology terms. Hypergeometric test and Benjamini & Hochberg False Discovery Rate correction were used to identify overrepresented GO terms at *p* = 5 % significance level (Benjamini and Yekutieli 2001). Cytoscape platform was used to visualize the results (Shannon et al. 2003). Variability in number and frequency of epitopes, possible antibody response and induced diseases have been analysed both on digested and non-digested proteins.

## Results

Altogether, 573 linear epitopes related to non-pollen type wheat allergies and celiac disease have been collected from IEDB, mainly originating from *Triticum aestivum*. A few epitopes have been identified from other *Triticeae* species, such as *Hordeum vulgare* and *Secale cereale*. As a result of the level of conservation analysis, 632 *Brachypodium* proteins were found that contained at least one epitope with 100 % sequence homology. Sequences were compared to developing *Brachypodium* seed ESTs and rice homologues. From the 632 proteins, 206 potential allergens were identified as being expressed in seed tissues.

The distribution of potential allergen proteins causing symptoms related to celiac disease and wheat allergies among the *Brachypodium* chromosomes showed that 81 % of the identified allergen proteins possessed celiac disease epitopes in their sequences (Table 1). Chromosome 1, 2 and 3 contained the majority of the toxic proteins, 33.5 % of the possible CD proteins were located on chromosome 1, followed by 23.4 % at chromosome 3 and 21 % at chromosome 2. Potentially allergen seed proteins have been digested with trypsin, pepsin and chymotrypsin using the PeptideCutter tool on Expasy. One hundred thirty-eight from the 167 celiac disease-related proteins were resistant to the digestion. Similarly, 63.83 % of the wheat allergy-related *Brachypodium* proteins remained intact after enzyme digestion (Table 1).

**Table 1:** Distribution of potential harmful proteins and epitopes encoded at the different *B. distachyon* chromosomes

Celiac disease					Wheat allergy			
Chromosome	Number of proteins	Epitopes found in *Brachypodium* seed proteins	Number of proteins containing intact epitopes when digested	Intact epitopes found in *Brachypodium* seed proteins	Number of proteins	Epitopes found in *Brachypodium* seed proteins	Number of proteins containing intact epitopes when digested	Intact epitopes found in *Brachypodium* seed proteins
1	56	FFQP	49	QQQP	11	AASVPE	5	AASVPE
		LQQQQQQQQQ		IPEQ		LQQQQQQQQQ		QQQQQQQQLQ
		QQQP		QQQQQQQQLQ		LTAASV		QQQPP
		IPEQ				PEAVLR		QQPPQQ
		QQQQQQQQLQ				QQQQQQQQLQ		
						QQQPP		
						QQPPQQ		
						REGKEV		
						VSALTG		
2	35	FFQP	21	PQQLPQ	20	AASVPE	12	LQQQQQQQQQ
		PQQLPQ		QQQP		ADINNE		AASVPE
		QQQP		IPEQ		GKEVLP		ADINNE
		IPEQ		LQQQQQQQQQ		LQQQQQQQQQ		QQQPP
		LQQQQQQQQQ				QQQPP		QQPPQQ
						QQPPQQ		
						VSALTG		
3	39	FFQP	33	QQQP	7	CRAMVK	6	GSQVPE
		QQQP		IPEQ		GSQVPE		QQQPP
		IPEQ				QQQPP		QQPPQQ
						QQPPQQ		
4	26	FFQP	24	QQQP	5	LQQQQQQQQQ	4	LQQQQQQQQQ
		QQQP		IPEQ		QQQPP		QQQPP
		IPEQ		LQQQQQQQQQ		QQPGQ		QQPGQ
		LQQQQQQQQQ						
5	11	QQQP	11	QQQP	4	QQQPP	3	QQQPP
		IPEQ		IPEQ		QQPPQQ		QQPPQQ
						SMLRSV		
*Σ*	167		138		47		30	

There were six CD-related epitopes present in this set of proteins: FFQQ, IPEQ, LQQQQQQQQQ, PQQLPQ, QQQP and QQQQQQQQLQ. The epitopes were identified either from alpha-gliadins or annotated as peptides originated from gluten proteins. Five of them were also found in the digested protein set.

Forty-seven proteins have been identified from the analysed protein pool of *B. distachyon* that have wheat allergen epitopes. Frequency of potential allergens was the highest at Chr 2 (42.6 %) and Chr 1 (23.4 %) (Table 1). These proteins possessed identity to 14 WA epitopes in their sequences. The epitopes, presented in Table 1, were originally identified from alpha gliadins, alpha-amylase inhibitors, omega gliadins, HMW glutenins and LMW glutenins, respectively. Epitopes such as AASVPE, ADINNE, GSQVPE, LQQQQQQQQQ, QQPGQ, QQPPQQ, QQQPP and QQQQQQQQLQ were resistant to digestion by all three enzymes.

**Fig. 1:**
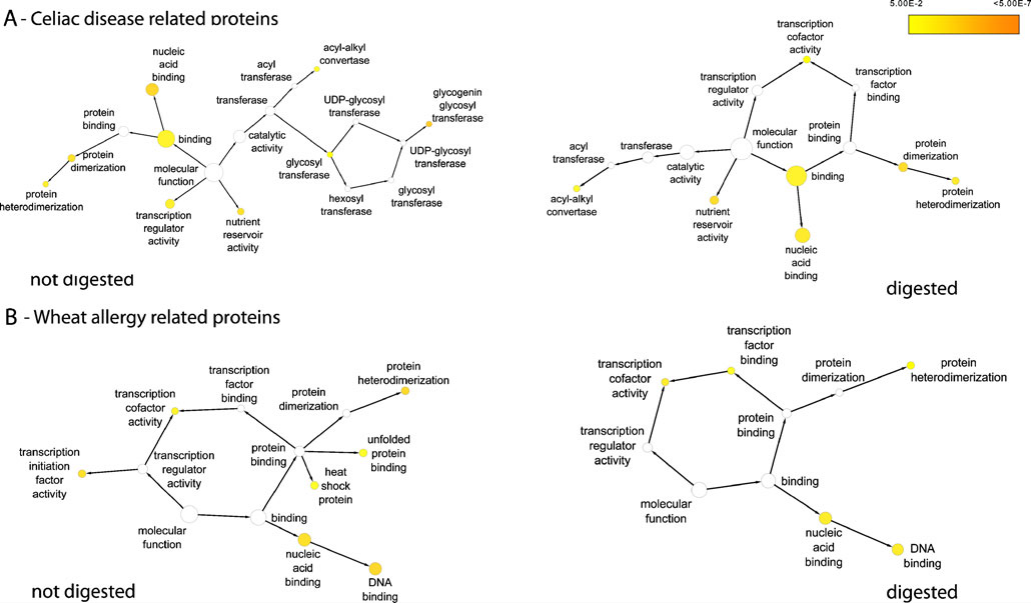
Molecular function of significantly overrepresented proteins in potential allergens in *B. distachyon*. **a** Maps of predominant molecular function terms in proteins containing celiac disease-related epitopes. **b** Maps of predominant molecular function terms in proteins possessing wheat allergy-related epitopes. Overrepresented Gene Ontology (GO) terms were assigned using Biological Networks Gene Ontology tool (BiNGO). Networks labelled with ‘not digested’ represent molecular functions of proteins with all the identified epitopes. ‘Digested’ label presents functions of proteins containing intact epitopes resistant to trypsin, pepsin and chymotrypsin digestion. The size of nodes is proportional to the number of genes assigned to the same GO term. Significantly overrepresented GO terms are labelled with coloured nodes. Colour scale of significant *p* values is presented

Large diversity in molecular function was found both in CD- and WA-related proteins based on the annotations in *Brachypodium* protein sequence database (version 1.2, http://www.brachypodium.org). In order to group the potentially allergen proteins into biological informative groups, their molecular functions have been characterized using sequence annotations and Gene Ontology terms. The BiNGO tool was used to find proteins with significantly overrepresented GO terms both in celiac disease- and wheat allergy-related proteins (Fig. 1). For detailed results of the BiNGO analysis, see the Electronic supplemental material (ESM) 1. Proteins containing CD epitopes fulfil molecular functions such as binding (protein and nucleic acid binding), catalytic activity, transcription regulator activity, nutrient reservoir activity and enzyme regulator activity. About 32 % of proteins could not be annotated with GO terms. Among proteins with known molecular functions, only 6 % of the CD-related proteins were assigned to nutrient reservoir functions (Fig. 1a and ESM 1). The number of potential wheat allergen proteins was 47, and 10 proteins could not be assigned to GO terms. Similar to CD-related proteins, these proteins also have primary functions in nucleic acid and proteins binding and also in transcription regulation (Fig. 1b).

## Discussion

The behaviour of food proteins during gastrointestinal passage depends on their primary structure, such as the presence and distribution of available amino acids recognized by the different enzymes. However, their folding strongly determines whether these proteins are exposed to the effect of different endopeptidases present in the stomach and the small intestines. Today, the number of available three-dimensional structure models for cereal seed proteins is extremely limited. Therefore, to consider inhibited digestive processes hindered by the unique structural characteristics, the following two extremes were used: Proteins without digestion represent a situation when all the cleavage sites were buried, while proteins digested with pepsin, trypsin and chymotrypsin simulate the case when all cleavage sites were exposed. Eighty-one percent of the identified allergen proteins were resistant to the enzymatic digestion. Based on these results, only 0.53 to 0.66 % of the entire *Brachypodium* proteome has the potential to cause symptoms related to cereal sensitivity. At the moment, we do not have information about the entire allergen content of the wheat seed compared to the wheat genome. However, about 90 % of the seed proteins consist of prolamins, based on which a higher amount of harmful proteins can be expected in wheat.

The typical length of polypeptides causing celiac syndrome is known to have 9–30 amino acids (Shan et al. 2002), and despite the fact that the identified *Brachypodium* epitopes have different length, based on their peptide sequence they can all provoke CD-related symptoms. When *Brachypodium* proteins were solely screened for the presence of celiac disease-related epitopes, the majority of hits showed only 80–90 % homology to the wheat CD epitopes (results not shown). Although this level of sequence homology can be indicative that these peptides might have some harmful effects, only epitopes with 100 % identical peptide sequences were considered in this analysis. However, the use of more than one platform for analysis, such as various allergy prediction methods and epitope mapping tools in combination with published or self-developed epitope databases, may reveal further relationships between protein sequences and related diseases and also a confirmation exercise in the decision-making process.

When potential allergens were assigned to gene ontology terms, one of the most remarkable results was the low-level nutrient reservoir activity. About 6 % of the CD-related proteins belong to storage proteins, while none of the WA proteins fulfilled storage activity. This is due to the low level of contribution of prolamin proteins to allergen proteins found in this study. Apparently, prolamins are not the major nutrient reserves in the *Brachypodium* seed. The proteins associated with nutrient reserve function in *B. distachyon* belong to avenin-like prolamins, or predicted proteins, but none of the gliadin- or glutenin-like prolamins has been identified. Avenins are storage proteins characteristic for oat; however, avenin-like proteins called farinins are also present as minor prolamins in wheat (Dupont et al. 2011). Most of the proteins with known molecular function belong to binding proteins, either as nucleic acid binding proteins or proteins involved in protein binding. Proteins with functions such as protein dimerization, heterodimerization and transcription factor binding also belong here in the group of binding proteins. Proteins with transcription regulator activity include bZIP transcription factors, transcriptional initiation factors, transcriptional co-repressors and proteins with zinc-finger domain. All these proteins contain several four to ten amino acid long polyglutamine stretches in their sequences, which might serve as toxic epitopes, similar to prolamin epitopes. Enzyme regulators include protein phosphatases and GTPases. Proteins with catalytic activity involve mainly acyl- and glycosyl-group transferases. The digested set of CD proteins is composed of a similar set of proteins, although the distribution of overrepresented functions is slightly modified (Fig. 1a). These protein families are involved in the regulation of different biological processes, such as regulation of flower development, postembryonic development and regulation of metabolic processes such as regulation of transcription.

The most striking difference between CD- and WA-related proteins analysed in this study was that there were no proteins assigned with storage protein function among the WA proteins. There was only one protein containing a peptide identical to an epitope originally found in omega gliadins. This protein shared about 40 % sequence homology with several alpha gliadins. Another group of proteins is involved in protein dimerization and hetero-dimerization activities or heat shock protein binding and unfolded protein binding. The majority of potential wheat allergens identified here has a role in nucleic acid binding, transcription factor binding and transcription regulation, and a large part of them possess zinc finger binding domains.

There are no studies reported yet on the immunoallergic characteristics of wheat proteins involved in transcriptional and translational regulations. This might be due to the relatively low number of related proteins present in mature seeds compared to some of the major allergens, such as gliadins. The common extraction techniques used for two dimension gel electrophoresis will likely fail when aiming for the detection of these proteins since they are low abundant, and the extraction of protein fractions enriched in e.g. transcription factors require specific extraction techniques. However, a predicted transcription factor with zinc-ion binding, APFI has been reported as IgE-binding protein (De Angelis et al. 2007). Similarly, proteins with zinc-ion binding capacity, such as plant homeodomain zinc finger proteins have been reported as major allergens for asthma and eczema (Rahman et al. 2010). Transcription factors with zinc fingers, heat shock-related proteins, different signal recognition receptors and different transporters were shown to be dominant in the soybean pollen transcriptome (Haerizadeh et al. 2009). This might suggest that transcription factors may possess some peptides in their sequence which results in similar IgE responses to some of the prolamin proteins. It is not known whether these transcription regulation-related proteins are present in the mature *Brachypodium* seed. Analysis of seed EST libraries may serve some information about their expression level. However, we do not have information on protein level if they are present in a detectable amount in dormant seeds. Proteome studies of nuclear extracts isolated from mature *Brachypodium* seeds will help to understand this question.

Detrimental effect of potential allergen proteins depends on many different genetic and environmental factors. These include elements influencing digestive and immunogenic mechanisms in the gastrointestinal tract. There are factors such as the amino acid composition, the tertiary structure and the expression levels of the toxic proteins that are primarily determined by the genetic and environmental effects of the allergen source. The bioinformatic tools used in allergen identification and allergen prediction have been extensively explored and reviewed by several research groups (Brusic et al. 2003, Tomar and De 2010). Generally, the following main approaches are used: sequence analysis, prediction and structural analysis (Brusic et al. 2003). The sequence analysis and comparison tools focus on the classification and characterization of potential allergens based on experimentally confirmed epitope sets and homolog allergens identified from related species (Brusic et al. 2003, Saha et al. 2006). Prediction tools are based on particular characteristics either in function or composition and chemical structure of proteins, based on which allergic potential can be identified (Brusic et al. 2003; Borges et al. 2007).

High-throughput genome sequencing is now one of the major methods in cereal allergy research to determine potential allergen epitopes. Although a number of epitopes may be false positives due to the complexity of human responses to food intake, the study presented here has several relevant outcomes. Our results emphasize the importance of considering the allergen characteristics of non-prolamin seed proteins for identifying new allergen protein families. The detected number of non-prolamin type allergen proteins is expected to increase when extensive seed proteomic studies, high-throughput bioinformatics and computational tools are involved simultaneously. The pleiotropic effects of utilizing technologies such as RNAi for tissue-specific allergenic-gene knockouts could lead to the compensation and over-expression of other proteins that may be high in glutamine and proline residues, and result in increased levels of allergic responses. These responses might be caused by protein families which do not normally show harmful effects. The outputs from our study indicate that the use of wild wheat species and primitive wheat lines can be a potential candidate for replacing the traditional ingredients of certain food products providing safety for people suffering from one or more of the health problem caused by wheat.

## Electronic supplementary material

Below is the link to the electronic supplementary material.

Supplemental Table 1 Results of BiNGO analysis, including percentage distribution of significantly overrepresented terms. Results for digested and non-digested sets of celiac disease- and wheat allergy-related proteins are presented in separate sheets. Terms with adjusted *p* values ≤5 % are significantly overrepresented (PDF 83 kb)
