# Supplementary material for: Brachypodium distachyon as a model for defining the allergen potential of non-prolamin proteins
Source: Funct Integr Genomics. 2012 Aug 30;12(3):439–46. doi: 10.1007/s10142-012-0294-z (PMC3431475; doi:10.1007/s10142-012-0294-z)
Supplement: Supplementary file 1 — Results of BiNGO analysis, including percentage distribution of significantly overrepresented terms. Results for digested and non-digested sets of celiac disease- and wheat allergy-related proteins are presented in separate sheets. Terms with adjusted p values ≤5 % are significantly overrepresented (PDF 83 kb) [file 10142_2012_294_MOESM1_ESM.pdf]

BiNGO analysis of proteins containing epitopes related to celiac disease without digestion with endopeptidases

Selected statistical test : Hypergeometric test
Selected correction : Benjamini & Hochberg False Discovery Rate (FDR) correction
Selected significance level : 0.05
Testing option : Use whole annotation as reference set
X: total number of genes in the analysed set
x: number of genes with identical GO term

GO term ID	N_CDnoDIG	n_CDnoDIG	X	x	description	p value	adjusted p value	%
8466	14990	4	100	3	glycogenin glucosyltransferase activity	1,1468E-06	0,00017317	2,702702703
3676	14990	3020	100	40	nucleic acid binding	4,0119E-06	0,0003029	36,03603604
46982	14990	21	100	4	protein heterodimerization activity	0,000010228	0,0003861	3,603603604
46983	14990	203	100	9	protein dimerization activity	8,4239E-06	0,0003861	8,108108108
45735	14990	98	100	6	nutrient reservoir activity	0,000048604	0,0014679	5,405405405
30528	14990	935	100	18	transcription regulator activity	0,00013906	0,0034997	16,21621622
3712	14990	18	100	4	transcription cofactor activity	0,00021857	0,0047149	3,603603604
5488	14990	10221	100	90	binding	0,00060388	0,011398	81,08108108
46912	14990	10	100	2	transferase activity, transferring acyl groups, acyl groups converted into alkyl on transfer	0,0019148	0,032126	1,801801802
30234	14990	293	100	7	enzyme regulator activity	0,0034536	0,04858	6,306306306
3677	14990	1809	100	23	DNA binding	0,003539	0,04858	20,72072072
8134	14990	54	100	3	transcription factor binding	0,0055852	0,070281	2,702702703
35251	14990	74	100	3	UDP-glucosyltransferase activity	0,013265	0,091274	2,702702703
46527	14990	87	100	3	glucosyltransferase activity	0,02039	0,10617	2,702702703
5515	14990	2265	100	22	protein binding	0,041585	0,16101	19,81981982
8194	14990	121	100	3	UDP-glycosyltransferase activity	0,04718	0,16962	2,702702703
16758	14990	362	100	3	transferase activity, transferring hexosyl groups	0,43585	0,59662	2,702702703
16757	14990	456	100	3	transferase activity, transferring glycosyl groups	0,59048	0,71905	2,702702703
16746	14990	294	100	2	transferase activity, transferring acyl groups	0,58693	0,71905	1,801801802
16740	14990	2876	100	18	transferase activity	0,65833	0,78895	16,21621622
3674	14990	14990	100	100	molecular_function	1	1	90,09009009
3824	14990	8089	100	36	catalytic activity	0,9999	1	32,43243243

BiNGO analysis of proteins containing epitopes related to celiac disease digested with endopeptidases

Selected statistical test : Hypergeometric test
Selected correction : Benjamini & Hochberg False Discovery Rate (FDR) correction
Selected significance level : 0.05
Testing option : Use whole annotation as reference set
X: total number of genes in the analysed set
x: number of genes with identical GO term

GO term ID	N_CDDIG	n_CDDIG	x	x	description	p value	adjusted p value	%
45735	14990	98	83	6	nutrient reservoir activity	1,68E-05	1,16E-03	7,228915663
46983	14990	203	83	8	protein dimerization activity	1,63E-05	1,16E-03	9,638554217
3712	14990	18	83	4	transcription cofactor activity	1,26E-04	5,79E-03	4,819277108
46982	14990	21	83	3	protein heterodimerization activity	2,03E-04	5,85E-03	3,614457831
3676	14990	3020	83	31	nucleic acid binding	2,12E-04	5,85E-03	37,34939759
5488	14990	10221	83	77	binding	6,41E-04	1,47E-02	92,77108434
46912	14990	10	83	2	transferase activity, transferring acyl groups, acyl groups converted into alkyl on transfer	1,32E-03	2,61E-02	2,409638554
8134	14990	54	83	3	transcription factor binding	3,31E-03	5,71E-02	3,614457831
30528	14990	935	83	12	transcription regulator activity	5,20E-03	7,64E-02	14,45783133
5515	14990	2265	83	20	protein binding	2,07E-02	1,19E-01	24,09638554
16746	14990	294	83	2	transferase activity, transferring acyl groups	4,87E-01	6,10E-01	2,409638554
16740	14990	2876	83	13	transferase activity	8,30E-01	9,19E-01	15,6626506
3674	14990	14990	83	83	molecular_function	1	1	100
3824	14990	8089	83	29	catalytic activity	1,00E+00	1	34,93975904

BiNGO analysis of proteins containing epitopes related to wheat allergies without digestion with endopeptidases

Selected statistical test : Hypergeometric test
Selected correction : Benjamini & Hochberg False Discovery Rate (FDR) correction
Selected significance level : 0.05
Testing option : Use whole annotation as reference set
X: total number of genes in the analysed set
x: number of genes with identical GO term

GO term ID	N_WAnoDIG	n_WAnoDIG	x	x	description	p value	adjusted p value	%
3677	14990	1809	37	15	DNA binding	1,10E-05	7,14E-04	40,54054054
46982	14990	21	37	3	protein heterodimerization activity	1,79E-05	7,14E-04	8,108108108
3676	14990	3020	37	18	nucleic acid binding	9,52E-05	1,90E-03	48,64864865
16986	14990	33	37	3	transcription initiation factor activity	7,18E-05	1,90E-03	8,108108108
3712	14990	18	37	2	transcription cofactor activity	8,85E-04	1,42E-02	5,405405405
51082	14990	123	37	3	unfolded protein binding	3,42E-03	4,56E-02	8,108108108
31072	14990	132	37	3	heat shock protein binding	4,17E-03	4,76E-02	8,108108108
8134	14990	54	37	2	transcription factor binding	7,83E-03	6,96E-02	5,405405405
30528	14990	935	37	7	transcription regulator activity	7,13E-03	6,96E-02	18,91891892
46983	14990	203	37	3	protein dimerization activity	1,36E-02	1,08E-01	8,108108108
5515	14990	2265	37	9	protein binding	9,55E-02	3,64E-01	24,32432432
5488	14990	10221	37	29	binding	1,22E-01	4,24E-01	78,37837838
3674	14990	14990	37	37	molecular_function	1	1	100

BiNGO analysis of proteins containing epitopes related to wheat allergies digested with endopeptidases

Selected statistical test : Hypergeometric test
Selected correction : Benjamini & Hochberg False Discovery Rate (FDR) correction
Selected significance level : 0.05
Testing option : Use whole annotation as reference set
X: total number of genes in the analysed set
x: number of genes with identical GO term

GO term ID	N_WADIG	n_WADIG	x	x	description	p value	adjusted p value	%
3712	14990	18	24	2	transcription cofactor activity	3,70E-04	6,78E-03	8,333333333
3677	14990	1809	24	10	DNA binding	2,52E-04	6,78E-03	41,66666667
3676	14990	3020	24	13	nucleic acid binding	2,31E-04	6,78E-03	54,16666667
46982	14990	21	24	2	protein heterodimerization activity	5,06E-04	6,96E-03	8,333333333
8134	14990	54	24	2	transcription factor binding	3,34E-03	3,68E-02	8,333333333
30528	14990	935	24	5	transcription regulator activity	1,47E-02	1,16E-01	20,83333333
46983	14990	203	24	2	protein dimerization activity	4,14E-02	2,28E-01	8,333333333
5488	14990	10221	24	20	binding	7,95E-02	2,92E-01	83,33333333
5515	14990	2265	24	5	protein binding	2,92E-01	5,94E-01	20,83333333
3674	14990	14990	24	24	molecular_function	1	1	100
